# Reduced vascular events in type 2 diabetes by biguanide relative to sulfonylurea: study in a Japanese Hospital Database

**DOI:** 10.1186/s12902-015-0045-y

**Published:** 2015-09-17

**Authors:** Makito Tanabe, Takashi Nomiyama, Ryoko Motonaga, Kunitaka Murase, Toshihiko Yanase

**Affiliations:** Department of Endocrinology and Diabetes Mellitus, Faculty of Medicine, Fukuoka University, Fukuoka, 814-0180 Japan

## Abstract

**Background:**

Some oral hypoglycemic agents (OHAs) have been suggested to reduce the risk of cardiovascular disease (CVD) in type-2 diabetes mellitus (T2DM). We ascertained if OHAs affect CVD risk in a cohort analysis of a multicenter medical-cost accounting database in Japan.

**Methods:**

Data of 4095 and 1273 T2DM patients in study 1 and study 2, respectively, were extracted from the database based on the following conditions: (i) began treatment with a single OHA (sulfonylurea, biguanide, thiazolidinedione, α-glucosidase inhibitor, glinide, or dipeptidyl peptidase-4 inhibitor) and continued the medication for ~1–1.4 years; (ii) hemoglobin (Hb)A1c level at baseline was available; (iii) age at baseline was 40–70 years; (iv) presence or absence of CVD history was not considered in study 1, but presence of CVD history was considered in study 2. Effects of OHAs relative to sulfonylurea on CVD risk according to ICD-10 were analysed using Kaplan-Meier curves during 104 weeks.

**Results:**

In study 1 targeting T2DM patients with and without a history of CVD, initial and baseline treatment with a biguanide significantly lowered the risk of CVD compared with that with a sulfonylurea, and was independent of HbA1c control. In study 2, a similar significant preventive effect of a biguanide on CVD risk relative to a sulfonylurea was observed in T2DM patients with history of CVD.

**Conclusions:**

Initial treatment and baseline treatment with a biguanide can reduce CVD risk relative to a sulfonylurea independent of the blood glucose-lowering effect of the biguanide in Japanese T2DM patients.

**Electronic supplementary material:**

The online version of this article (doi:10.1186/s12902-015-0045-y) contains supplementary material, which is available to authorized users.

## Background

Subjects with diabetes mellitus (DM) carry a higher risk of cardiovascular disease (CVD) than healthy individuals. A total of 40.4 % of individuals with type-2 DM (T2DM) die from CVD [1]. In an observational study in Finland, the prevalence of myocardial infarction (MI) in T2DM patients without a history of MI and in non-T2DM patients with a history of MI was similar (20.2 and 18.8 %, respectively) [2]. In Japan, the relative risk of coronary heart disease or cerebral infarction has been reported to be 3.0 compared with subjects with normal tolerance to glucose [3]. If MI occurs in a patient with DM, the risk of future mortality can increase by up to 4.6-fold compared with patients without DM without a history of MI [4]. However, the risk of CVD cannot be predicted completely by measuring levels of fasting blood glucose or hemoglobin (Hb)A1c, which is usually measured to observe the efficacy of anti-DM drugs for lowering glucose levels in blood. Several clinical trials have attempted to examine the incidence of CVD as a feature of drug efficacy [5–7].

Roumie et al. analyzed the efficacy of oral hypoglycemic agents (OHAs) for the prevention of cardiovascular events. They reported that metformin showed a significant reduction in the prevalence of death compared with a sulfonylurea (SU) [5]. The Study to Prevent Non-Insulin Dependent Diabetes Mellitus (STOP-NIDDM) revealed that the incidence of cardiovascular events or hypertension was reduced significantly in patients using one type of α-glucosidase inhibitor (α-GI): acarbose [7]. Meta-analyses of seven clinical trials focusing on acarbose showed similar results [8]. The thiazolidinedione (TZD) pioglitazone has been shown to slow atherosclerosis in T2DM patients as estimated by progression of maximal carotid intima-media thickness [9] and coronary atherosclerosis [10] compared with glimepiride. Glucagon-like peptide (GLP)-1-based therapy, which includes dipeptidyl peptidase (DPP)-4 inhibitors and GLP-1 receptor agonists, has become a popular treatment for patients with T2DM. The DPP-4 inhibitor sitagliptin has been speculated to lower the prevalence of CVD [11]. However, recent large-scale studies reported that, among patients with T2DM who had experienced acute coronary syndrome recently, the prevalence of major adverse cardiovascular events was not increased or decreased with the DPP-4 inhibitors saxagliptin [12] or alogliptin [13], as compared with placebo despite better control of HbA1c levels with both DPP-4 inhibitors than with placebo. Even if inconclusive, these results suggest that some OHAs may have beneficial effects in reducing CVD events in individuals with T2DM.

The actual treatment goal of HbA1c by OHAs is recommended to be 7.0 % (NGSP; National Glycohemoglobin Standardization Program) for the prevention of diabetic complication in Japan [14]. There are numerous difficulties in assessment of the reduction of cardiovascular risk by OHAs because a large volume of patient information must be collected. Moreover, few reliable medical databases are available in Japan, so data analyses are difficult. Consequently, in Japan, there is little evidence regarding reduction in cardiovascular risk by OHAs. One way to solve this problem may be to extract information from the National Health Insurance program in Japan. This program offers universal health coverage for all citizens (including comprehensive medical services and prescription medicines) and covers >99 % of Japanese citizens.

Previously, we investigated the utility of this type of multicenter medical-cost accounting database from T2DM patients in 15 hospitals (Medical Data Vision Co., Ltd. (MDV), Tokyo, Japan) by retrospective analyses. Statistical data of subjects showed an almost identical tendency with that of Patient Survey 2008 conducted by the Ministry of Health, Labour and Welfare in Japan (MHLWJ; estimated number of subjects = 2,386,000), including the ratio of patients according to sex, age distribution, and complications (e.g., dyslipidemia, hypertension). These findings suggested that the MDV database could reflect the current situation of T2DM in Japan [15]. Actually, the increased risk of acute pancreatitis in patients with T2DM using the MDV database has been reported recently [16]. In the present study, we wanted to ascertain if differences in reduction of the risk of CVD among various OHAs could be observed using the MDV database.

## Methods

### Source of data

The study protocol was approved by the Review Board on Clinical Research of Fukuoka University (Fukuoka, Japan). This retrospective observational study was done using a hospital-based composite database stored in hospital electronic-information systems constructed by MDV. MDV data were purchased by FUJIFILM Pharma Co., Ltd. A contract between FUJIFILM Pharma Co., Ltd., and Fukuoka University Hospital enabled analyses to be carried out. The current database of MDV has been expanded to a much greater extent than that reported previously [15]. The database of the medical information of patients (including laboratory data) was extracted from the medical-cost accounting system of 103 institutions (19 hospitals with <200 beds; 66 hospitals with 200–499 beds; and 18 hospitals with ≥500 beds) in Japan. This database contains information about age, sex, diagnosis, International Classification of Diseases (ICD)-10 code, surgical history, outpatient/inpatient status, prescriptions, and laboratory data. Written informed consent was not obtained from each patient because all data were extracted from the database retrospectively. Nevertheless, patient anonymity was guaranteed.

### Population

Two studies (study 1 and study 2) were undertaken. The population of patients extracted in the present study satisfied the following conditions: (i) began treatment with a single OHA from 1 April 2008 to 30 April 2013; (ii) HbA1c level (NGSP) at baseline was available; (iii) age at baseline was 40–70 years; (iv) the presence or absence of CVD history was not considered in study 1, but the presence of CVD history was considered in study 2.

**Fig. 1:**
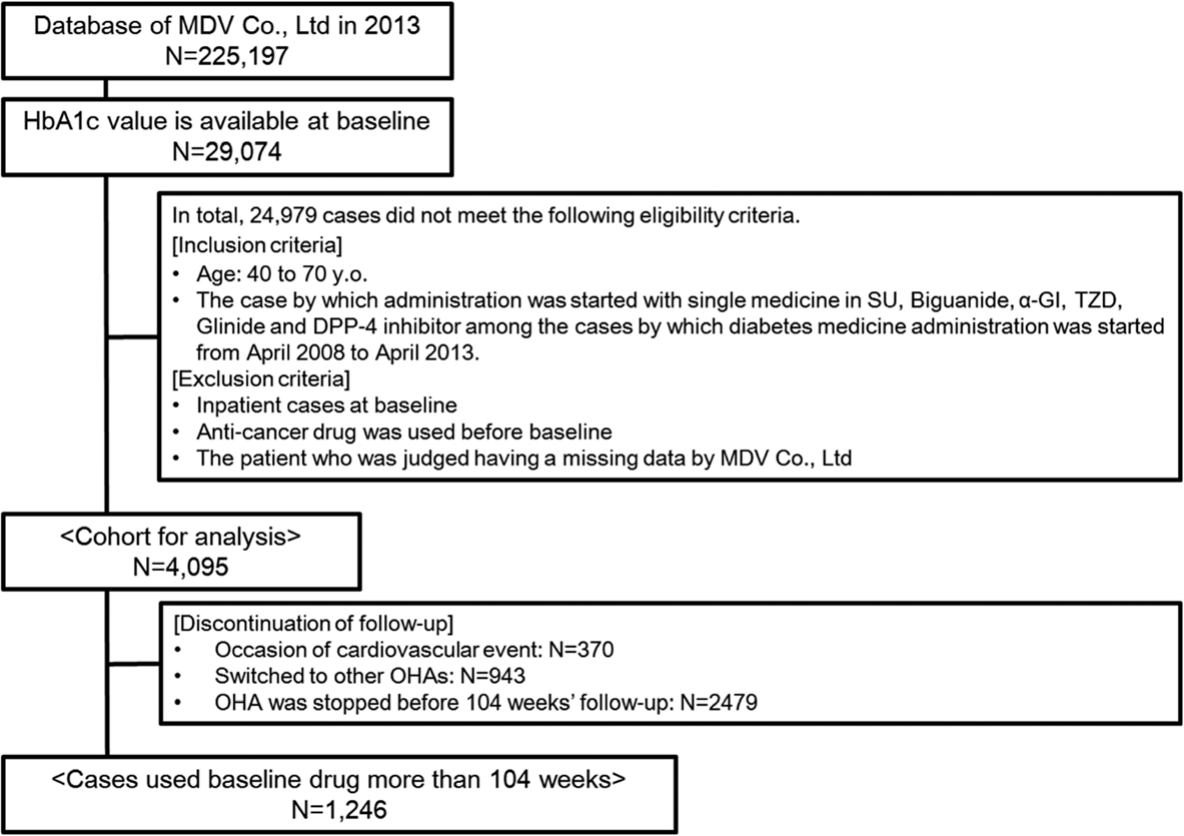
Outline and flowchart of the protocol for patient selection for analyses of cardiovascular events in study 1

**Fig. 2:**
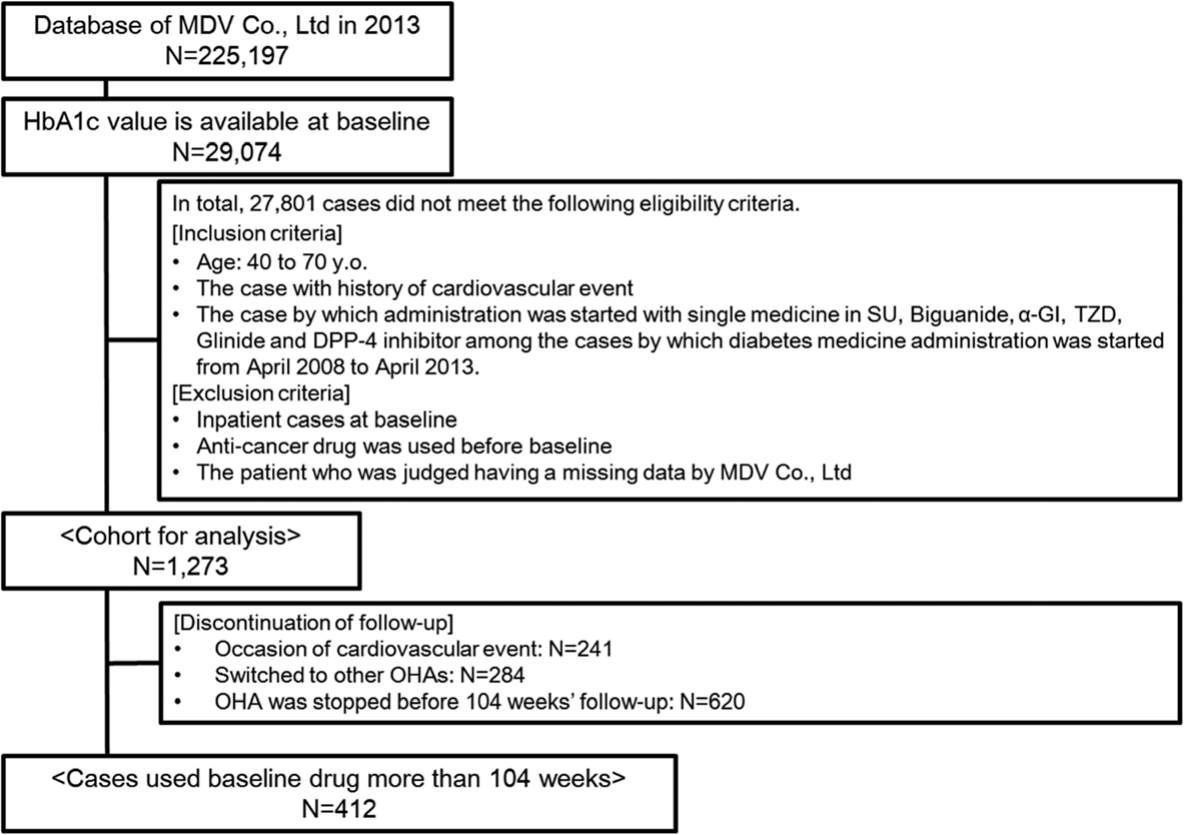
Outline and flowchart of the protocol for patient selection for analyses of cardiovascular events in study 2

Exclusion criteria were: (i) began treatment with insulin, GLP-1 analog, or compounding agent at baseline; (ii) began treatment with ≥2 types of OHA; (iii) admitted to hospital at baseline; (iv) an anti-cancer drug was used before baseline measurements were taken; (v) The patient who was judged having a missing data by MDV Co., Ltd. The MDV database in 2013 contained the data of 225,197 individuals. Among them, the baseline value of HbA1c was available for 29,074 patients. After careful adherence to inclusion and exclusion criteria, 4095 and 1273 individuals were subjected to cohort analyses of study 1 and study 2, respectively. Outlines as well as the classification of inclusion and exclusion in study 1 and study 2 are summarized in Figs. 1 and 2. Patients with cancer or liver cirrhosis were not listed in any drug groups. However, renal failure was noted in study 1: 2/629 (0.3 %) in the SU group, 4/1305 (0.3 %) in the BG group, 2/592 (0.3 %) in the α-GI group, 2/351 (0.6 %) in the TZD group, 2/223 (0.9 %) in the glinide group, and 6/995 (0.6 %) in the DPP-4 inhibitor group. Renal failure was also noted in study 2: 1/228 (0.4 %) in the SU group, 0/325 (0 %) in the BG group, 0/203 (0 %) in the α-GI group, 1/140 (0.7 %) in the TZD group, 2/70 (2.9 %) in the glinide group, and 6/307 (2.0 %) in the DPP-4 inhibitor group. However, there was no significant difference in the involvement of renal failure among the above 6 groups by Chi-squared test, suggesting little impact on the analysis. So we retained these data for the analysis.

### Definition of a cardiovascular event

Information regarding cardiovascular events was obtained from the MDV database. A cardiovascular event was defined as suffering angina pectoris (codes I200, I201, I208 and I209), MI (I210–I213, I219, I220, I221 and I229), heart failure (I500, I501 and I509), cerebral infarction (I630–I635, I638 and I639), cerebral hemorrhage (I614 and I619), or subarachnoid hemorrhage (I600–I602, I604, I605 and I609).

### Follow-up study

Observation was stopped at the time of: (i) a cardiovascular event; (ii) changing of the OHA at baseline; (iii) discontinuation of the drug at baseline before a prescription period of 2 years. However, observation was not suspended if a new OHA was added to the first drug. Finally, 1246 subjects and 412 cases in study 1 and study 2, respectively, were treated continuously by a baseline drug without a cardiovascular event being recorded for >2 years (summarized in Figs. 1 and 2).

### Data analyses

Baseline characteristics of patients (sex, age, HbA1c level, use of other drugs (anti-hypertension or anti-dyslipidemia)) and cardiovascular events were analyzed. Subjects were divided into six groups according to the baseline drug. Prevalence of CVD and change in HbA1c level from baseline in each drug group was assessed.

### Statistical analyses

To verify the independence of the analysis in the present study, statistical evaluation was undertaken by a specialist organization (ING Corp., Tokyo, Japan). ING Corp., are in no way associated with our institution or any drug company (including FUJIFILM Pharma Co., Ltd.). Statistical analyses were done using SAS v9.1.3 for Windows (SAS Institute, Cary, NC, USA). Prevalence of CVD in each group was investigated by Kaplan-Meier analyses. Comparisons between groups were achieved using the Cox-proportional hazard model adjusted for sex, age, HbA1c level (NGSP), use or non-use of anti-hypertensive drugs, and use or non-use of anti-dyslipidemia drugs. For consideration of the type-1 error in the Student’s *t*-test, patient characteristics at baseline between two groups (SU *vs* BG, SU *vs* α-GI, SU *vs* TZD, SU *vs* glinide, and SU *vs* DPP-4 inhibitor) were compared by the Dunnett test. *P* < 0.05 was considered significant.

## Results

### Validity of the database

The Japanese Patient Survey of 2011 for patients with DM was published by the MHLWJ. Hence, we compared our extracted database from 2013 with that of the MHLWJ. Our database contained 225,197 subjects and the percentage of males was 60.7 %. Based on data from the MHLWJ, the estimated number of subjects with DM in Japan was 2,700,000, and the percentage of males was 55.1 %. Age distribution of subjects with DM in our database was 5.8 % in their forties, 13.8 % in their fifties, 28.6 % in their sixties, 29.9 % in their seventies, and 15.8 % in their eighties, whereas that for the MHLWJ was 5.6, 13.7, 32.4, 31.1, and 13.4 %, respectively. The percentage of males in our database was 71.5 % in their forties, 68.7 % in their fifties, 65.6 % in their sixties, 60.2 % in their seventies, and 47.3 % in their eighties, whereas that of MHLWJ was 68.2, 61.2, 57.8, 53.8 and 40.9 %, respectively. These results suggested that our database could reflect the current situation of DM in Japan, as reported previously.

### Baseline characteristics of patients

Baseline characteristics of 4095 subjects in study 1 and 1273 subjects in study 2, including the six groups based on the type of OHA (i.e., SU, biguanide, α-GI, TZD, glinide, DPP-4 inhibitor) are shown in Tables 1 and 2, respectively. Mean age of biguanide and TZD groups was slightly (but significantly) younger than the SU group in study 1 and study 2. Treatment with anti-hypertension and anti-dyslipidemia drugs was undertaken in all OHA groups. The detailed classification of antihypertensive drugs and anti-dyslipidemia drugs used in subjects of study 1 and 2 are shown in Additional files 1 and 2, respectively. Mean HbA1c levels (NGSP) of biguanide, α-GI, TZD and glinide were slightly (but significantly) lower than those of the SU group in study 1 and study 2. Prevalence of patients with a high HbA1c value (≥6.5 %) was not significantly different between the SU group and the other groups in study 1 and study 2. Mean duration of administration of baseline drugs was 320–457 days in study 1 and 330–540 days in study 2. In study 1, the duration of administration of BG, α-GI, TZD and glinide was not significantly different from that of SU, but that of DPP-4 inhibitor was significantly shorter than that of SU. In study 2, the duration of administration of BG was significantly longer than that of SU, and that of DPP-4 inhibitor was significantly shorter than that of SU.

**Table 1:** Clinical profiles of T2DM patients treated with oral hypoglycemic agents (OHAs) in study 1

		SU	BG		α-GI		TZD		Glinide		DPP-4 inhibitor	
		*N* = 629	*N* = 1305	*P*-value	*N* = 592	*P*-value	N = 351	*P*-value	*N* = 223	*P*-value	*N* = 995	*P*-value
	Age	62 (60.8 ± 7.1)	59 (58.0 ± 7.9)	<0.001	61 (60.1 ± 7.3)	0.38	60 (59.5 ± 7.2)	0.044	61 (60.4 ± 6.9)	0.95	62 (60.0 ± 7.6)	0.15
	Male	454 (72.2)	823 (63.1)	-	393 (66.4)	-	236 (66.4)	-	140 (62.8)	-	634 (63.7)	-
Anti-hypertensive agent and anti-dyslipidemia agent	None	181 (28.8)	486 (37.2)	-	176 (29.7)	-	93 (26.5)	-	79 (35.4)	-	310 (31.2)	-
	Both of them	174 (27.7)	318 (24.4)	-	212 (25.8)	-	115 (32.8)	-	51 (22.9)		302 (30.4)	-
	One of them	274 (43.6)	501 (38.4)	-	204 (34.5)	-	143 (40.7)	-	93 (41.7)	-	383 (38.5)	-
HbA1c (NGSP %)		7.4 (7.9 ± 1.7)	7.2 (7.7 ± 1.6)	0.004	6.7 (6.9 ± 1.2)	<0.001	6.7 (6.9 ± 1.2)	<0.001	7.0 (7.1 ± 1.3)	<0.001	7.4 (7.8 ± 1.5)	0.90
	Minimum	4.7	5.0		4.9		5.1		4.6		5.1	
	Maximum	14.5	15.1		15.5		12.2		15		17.7	
	<6.5 %	100 (15.9)	216 (16.6)		224 (37.8)		125 (35.6)		60 (26.9)		89 (8.9)	
				-		-		-		-		-
	6.5% ≤	529 (84.1)	1089 (83.5)		369 (62.2)		226 (64.4)		163 (73.1)		906 (91.1)	
Duration of administration of baseline drug (days)		537 (457 ± 282)	612 (476 ± 278)	0.45	486 (453 ± 273)	1.00	521 (473 ± 249)	0.86	575 (499 ± 246)	0.16	281 (320 ± 241)	<0.001

Data are n(%) or median (mean ± standard deviation). *P*-value: Dunnett’s test for SU

**Table 2:** Clinical profiles of T2DM patients treated with oral hypoglycemic agents (OHAs) in study 2

		SU	BG		α-GI		TZD		Glinide		DPP-4 inhibitor	
		*N* = 228	*N* = 325	*P*-value	*N* = 203	*P*-value	N = 140	*P*-value	*N* = 70	*P*-value	*N* = 307	*P*-value
Age		63.5 (62.4 ± 6.3)	61 (60.8 ± 6.7)	0.025	63 (61.3 ± 6.9)	0.27	62 (60.5 ± 6.7)	0.028	62 (61.1 ± 6.1)	0.50	63 (61.6 ± 6.8)	0.52
Male		183 (80.3)	206 (63.4)	-	152 (74.9)	-	101 (72.1)	-	50 (71.4)	-	216 (70.4)	-
Anti-hypertensive agent and anti-dyslipidemia agent	None	24 (10.5)	49 (15.1)	-	21 (10.3)	-	20 (14.3)	-	11 (15.7)	-	34 (11.1)	-
	Both of them	109 (47.8)	141 (43.4)	-	126 (62.1)	-	67 (47.9)	-	30 (42.9)	-	166 (54.1)	-
	One of them	95 (41.7)	135 (41.5)	-	56 (27.6)	-	53 (37.9)	-	29 (41.4)	-	107 (34.9)	-
HbA1c (NGSP %)		7.3 (7.6 ± 1.5)	6.9 (7.1 ± 1.1)	<0.001	6.6 (6.7 ± 1.0)	<0.001	6.5 (6.8 ± 1.2)	<0.001	6.9 (6.9 ± 0.9)	<0.001	7.3 (7.6 ± 1.3)	0.97
	Minimum	4.7	5.4		4.9		5.1		4.8		5.1	
	Maximum	14.1	14.4		11.4		11.8		9.4		13.7	
	<6.5%	44 (19.3)	78 (24.0)		87 (42.9)		62 (44.3)		21 (30.0)		29 (9.4)	
				-		-		-		-		-
	6.5 % ≤	184 (80.7)	24.7 (76.0)		116 (57.1)		78 (55.7)		49 (70.0)		278 (90.6)	
Duration of administration of baseline drug (days)		616 (477 ± 279)	730 (540 ± 261)	0.022	700 (510 ± 263)	0.56	596.5 (499 ± 251)	0.90	524.5 (485 ± 251)	1.00	302 (330 ± 245)	<0.001

Data are n(%) or median (mean ± standard deviation). *P*-value: Dunnett’s test for SU

### Prevalence of cardiovascular events in each group

Detailed data on the prevalence of cardiovascular events in study 1 and study 2 are shown in Table 3A and B, respectively. Based on these data, composite (cardiac and cerebrovascular) cardiovascular events were used mainly for statistical analyses subsequently because meaningful values could not be obtained in the analysis of cerebral events only due to the small number of events. The number of cardiovascular events (1000 person-years) is shown in Table 4A and B.

**Table 3:** Number of cardiovascular events in subjects in study 1 (A) and study 2 (B)

OHAs (Administration of one medicine)	Cardiovascular events (patients)	Cardiac events (patients)	Stroke (patients)	Analysis
(A)				
SU	80	71	10	629
Biguanide	84	76	10	1305
α-GI	63	58	7	592
TZD	35	29	6	351
Glinide	21	19	3	223
DPP-4 inhibitor	87	80	8	995
(B)				
SU	58	53	6	228
Biguanide	47	48	4	325
α-GI	40	40	3	203
TZD	26	24	3	140
Glinide	14	14	1	70
DPP-4 inhibitor	56	57	3	307

Cardiovascular event: angina pectoris, myocardial infarction, heart failure, cerebral infarction, cerebral hemorrhage and subarachnoid hemorrhage

Cardiac event: angina pectoris, myocardial infarction and heart failure

Stroke event: cerebral infarction, cerebral hemorrhage and subarachnoid hemorrhage

**Table 4:** Number of cardiovascular events (1000 person-years) in subjects in study 1 (A) and study 2 (B)

	N	The number of cardiovascular events	1000 person-years
(A)			
SU	629	80	108.9
BG	1305	84	51.0
α-GI	592	63	91.3
TZD	351	35	82.3
Glinide	223	21	71.2
DPP-4 inhibitor	995	87	105.2
(B)			
SU	228	58	224.0
BG	325	47	106.2
α-GI	203	40	159.4
TZD	140	26	154.7
Glinide	70	14	162.1
DPP-4 inhibitor	307	56	222.7

**Fig. 3:**
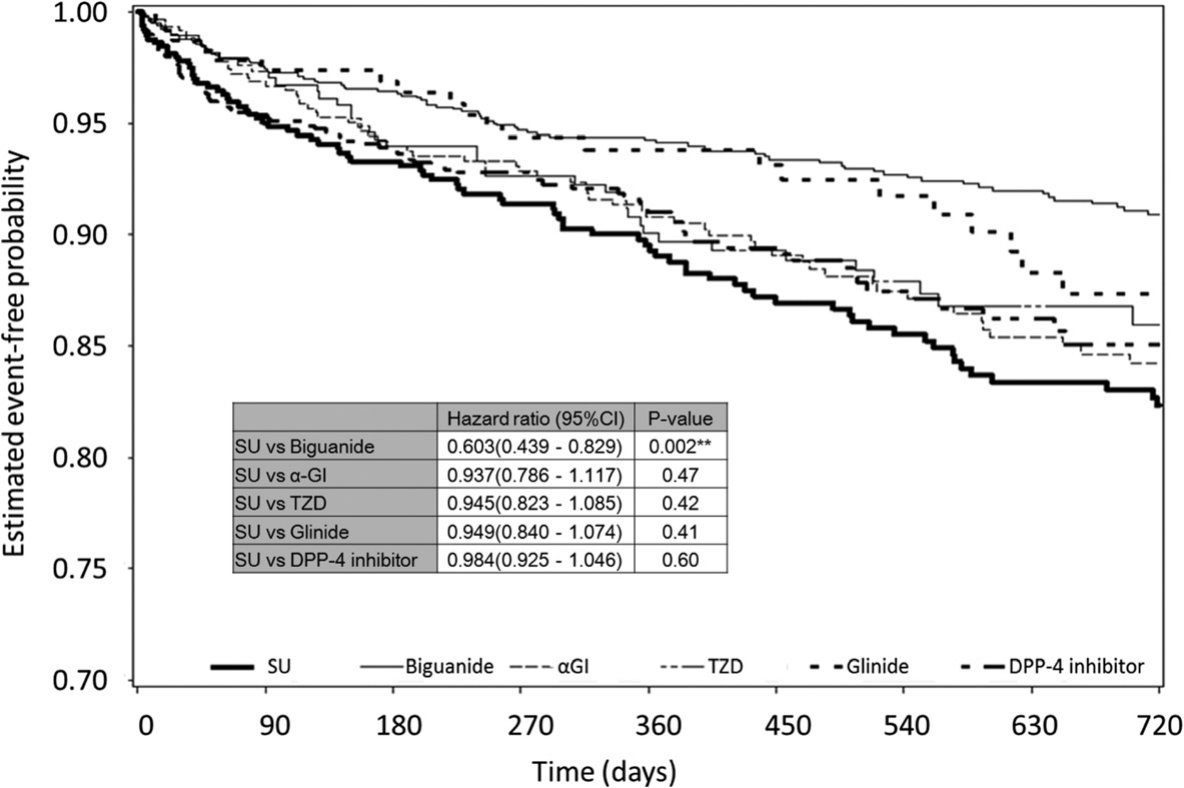
Kaplan-Meier curve for cardiovascular events during 104 weeks in patients with T2DM with or without a history of cardiovascular events. Number of patients in each drug group is listed in Table 1. Numbers of those in which 104-week follow-up was completed were 629 in SU, 1305 in biguanide, 592 in α-GI, 351 in TZD, 223 in glinide, and 995 in DPP-4 inhibitor groups, respectively (Table 1). Numbers of those in which a cardiovascular event was observed were 80 in SU, 84 in biguanide, 63 in α-GI, 35 in TZD, 21 in glinide, and 87 in DPP-4 inhibitor groups, respectively (Table 3A). Statistical comparison of cardiovascular events during 104 weeks in patients with T2DM with a history of cardiovascular events is shown in the inserted small table. Hazard ratio, Cox proportional hazard model adjusted by sex, age, HbA1c value (NGSP), use or non-use of anti-hypertensive drugs, use or non-use of anti-dyslipidemia drugs. CI indicates a 95 % confidence interval. **P < 0.01

Kaplan-Meier curves for T2DM patients who experienced ≥1 cardiovascular event in each group and statistical comparisons between SU and other drugs in study 1 are shown in Fig. 3. For determination of the hazard ratio (HR), the Cox proportional hazard model was adjusted for sex, age, HbA1c value (NGSP), use or non-use of anti-hypertensive drugs, and use or non-use of anti-dyslipidemia drugs. During a 104-week observation period, patients who began treatment with BG showed a significantly lower risk of cardiovascular events compared with those who started treatment with SU. HR of BG relative to SU was 0.603 (95 % confidence interval [CI], 0.439–0.829, *P* = 0.002). Significant risk reduction by BG relative to SU was also observed in analyses of cardiac events only (HR, 0.613; 95 % CI, 0.438–0.857; *P* = 0.004). However, α-GI, TZD, glinide and DPP-4 inhibitor did not show such significant reduction in risk compared with SU in terms of cardiovascular events and cardiac events (data not shown). A similar analysis using Kaplan-Meier curves targeting T2DM patients who did not have a history of cardiovascular events (*n* = 2822) did not show a significant difference in the prevalence of cardiovascular events between SU and other OHAs (data not shown).

Kaplan-Meier curves for T2DM patients who had history of CVD events (study 2) revealed that the BG group also significantly reduced the risk of cardiovascular events relative to the SU group. The Cox proportional hazard model was adjusted for sex, age, HbA1c value (NGSP), use or non-use of anti-hypertensive drugs, and use or non-use of anti-dyslipidemia drugs, and HR was determined to be 0.566 (95 % CI, 0.376–0.852, *P* = 0.006). However, a significant reduction in risk by BG relative to SU was not observed if analyses were restricted to cardiac events or stroke events (data not shown). With regard to all other OHAs, significance was not observed in the risk reduction of cardiovascular events, cardiac events, or stroke events (data not shown).

### Changes in HbA1c levels during the observation period

Changes in HbA1c levels from baseline (0 week) to 2 years (104 weeks) in patients in whom a cardiovascular event was observed were compared among OHAs. In patients who began treatment with SU, BG, α-GI, a TZD, glinide or DPP-4 inhibitor, the HbA1c level was reduced with SU, BG, α-GI, a TZD and DPP-4 inhibitor, but was increased with glinide. The maximum lowering effect in HbA1c level was observed in the DPP-4 inhibitor group. The difference in the change in HbA1c level between SU (−0.31 ± 0.07, *N* = 236) and glinide (0.08 ± 0.12, *N* = 81), and between SU and DPP-4 inhibitor (−0.75 ± 0.11, *N* = 95) was significant (*P* = 0.0005 and *P* < 0.0001, respectively). However, the extent of change in the level of BG (−0.30 ± 0.05, *N* = 542), α-GI (−0.21 ± 0.08, *N* = 195) and TZD (−0.31 ± 0.11, *N* = 92) relative to that of SU was not significant (*P* = 0.90, *P* = 0.37 and *P* = 0.95, respectively). Similar results were observed in study 2. The difference in the change in HbA1c level between SU (−0.43 ± 0.11, *N* = 80) and TZD (−0.003 ± 0.16, *N* = 42) and between SU and glinide (0.50 ± 0.20, *N* = 24) was significant (*P* = 0.029 and *P* < 0.001, respectively). However, the extent of change in the level of BG (−0.19 ± 0.08, *N* = 159), α-GI (−0.19 ± 0.11, *N* = 80) and DPP-4 inhibitor (−0.27 ± 0.20, *N* = 27) relative to that of SU was not significant (*P* = 0.069, *P* = 0.12 and *P* = 0.48, respectively).

## Discussion

In the present study, T2DM patients who started treatment with BG showed a significantly lower prevalence of cardiovascular events compared with those who started treatment with SU. This phenomenon was unrelated to the presence or absence of CVD history.

Our results are based on a comparison of OHAs with SU, and do not suggest that cardiovascular risk is reduced by BG but increased with SU. There were slight differences in the backgrounds of patients in the six OHA groups (e.g., slightly lower mean age and HbA1c level in the BG group compared with the SU group). For determination of the HR, the Cox proportional hazard model was adjusted for sex, age, HbA1c value (NGSP), use or non-use of anti-hypertensive drugs, and use or non-use of anti-dyslipidemia drugs. As a result, 2-year (104-week) observation of patients with T2DM (age, 40–70 years) who began treatment with a single OHA revealed that BG significantly reduced the prevalence of cardiovascular events compared with SU.

Meta-analyses focusing on the efficacy of BG for a reduction in cardiovascular risk have been reported [17]. In that report, metformin (a type of BG) reduced the incidence of CVD compared with placebo/non-treated subjects. Survival was also favorable in the metformin group, but was reduced if metformin was administered with SU. Another report made a direct comparison between BG and SU in which metformin reduced the incidence of cardiovascular events and mortality [5]. The results of the present study are in accordance with the results of those reports. Compared with SU, BG has been reported to have favorable effects on body weight, lipid levels, and blood pressure [18]. BG has also been reported to reduce the activity of plasminogen activator inhibitor-1, which is a factor for thrombogenesis [19] and exerts its protective effect against ischemia-reperfusion injury after MI. These effects are considered not to be explained fully by the lowering effect of blood glucose [20]. Some SU drugs bind to the SUR_2A/B_ receptor (located in the myocardium and smooth muscle cells in coronary arteries) and reduce the effect of ischemic preconditioning, and possibly increase the risk of cardiovascular events [21]. Thus, the risk reduction of cardiovascular events by BG relative to SU in the present study can be accounted for (at least in part) by the mechanisms of action of BG and SU stated above. Namely, a possibility of increased risk of CV events by SU and a possibility of reduced risk of CV evenyts by BG may result in our results.

In our analyses, the reduction in cardiovascular risk between drugs relative to SU was not associated with the reduction in HbA1c level. Several clinical trials, including Action to Control Cardiovascular Risk in Diabetes (ACCORD) [22], Action in Diabetes and Vascular Disease (ADVANCE) [23] and Veterans Affairs Diabetes Trial (VADT) [24], have shown that intensive control of glycemia does not consistently reduce the prevalence of cardiovascular events and, in some cases, may be harmful. One reason for the failure in the reduction in the prevalence of cardiovascular events is speculated to be hypoglycemia [25]. Hypoglycemia is associated with increased activation of the sympathetic nervous system, which has been suggested to be a major risk factor for adverse events [23]. Although not observed as often as that seen with insulin, SU carries a relatively greater risk of hypoglycemia [26]. This phenomenon may explain (at least in part) the relative increase in the prevalence of cardiovascular events in the SU group compared with BG groups even though a similar degree of reduction in the HbA1c level was achieved.

Our study had several important limitations. First, despite being a population-based study using a hospital database, the sample size for analysis was not very large because the inclusion and exclusion criteria of subjects had to be considered. Second, this study was not a prospective type of cohort analysis, making correct evaluation of the clinical impact of each OHA very difficult. Even though the first OHA was continued as the baseline drug for ~1–1.4 years according to mean values, the first OHA was sometimes switched to other OHAs, used with other OHAs from certain time-points, or stopped before 104 weeks. Third, the duration of follow-up (2 years) may be relatively short for comparison of the prevalence of cardiovascular events. Fourth, information of antihypertensive drugs, antiplatelet or anticoagulant medications and anti-dyslipidemia drugs which can affect CV outcomes, were partly but not fully available from the database. We could not completely rule out a possibility that these drugs might have some impact on the CV outcome in this study.

In the present study, the effect of α-GI on reduction in cardiovascular risk was not favorable compared with that for SU, a result that is in contrast with other clinical studies [7, 8]. α-GI might have had little impact on the prevalence of cardiovascular events because of its relatively lower level of HbA1c at the start of our study. The vascular-protective effects of TZD or DPP-4 inhibitor relative to SU was not demonstrated in our study despite some favorable evidence in recent years [9–11, 27, 28], though this topic remains controversial [12, 13]. DPP-4 inhibitors are effective and safe [29] and now expanding the OHA market in Japan enormously, which will enable this type of population-based analyses to be undertaken again. Hence, the vascular-protective effect of OHAs (including α-GI and DPP-4 inhibitor) seems to be inconclusive and needs more careful interpretation in the context of the limitations of our study. A slightly shorter duration of administration of DPP-4 inhibitor relative to SU in our study might have affected the results. Nevertheless, we can say with confidence that initial treatment with BG and its baseline treatment for a certain period of time could aid prevention of CVD events in T2DM patients in Japan.

Despite the study limitations detailed above, our study analyzing a database containing information on Japanese individuals with T2DM in 103 hospitals revealed that first treatment and baseline treatment for a certain duration with BG could significantly reduce the prevalence of cardiovascular events compared with SU, and that this effect was independent of its blood glucose-lowering effect. The American Diabetes Association and European Association for the Study of Diabetes issued guidelines [30] that incorporated information from clinical trials focusing on cardiovascular outcomes [22–24]. Those guidelines recommend initial treatment with BG and emphasize a patient-centric approach to treatment rather than adherence to stricter control of glycemia [29]. BG does not have strong glucose-lowering effects, but our findings will aid the choice of anti-DM drugs for the prevention of cardiovascular events beyond glycemic control.

## Conclusions

In Japanese subjects with T2DM, initial treatment and baseline treatment with BG can reduce CVD risk relative to that of SU, and this effect is independent of the blood glucose-lowering effect of BG.

## Availability of data and materials

Not applicable.

## Additional files

Additional file 1:
**Details of antihypertensive drugs and** **anti-dyslipidemia** **drugs used for subjects in study 1.** (PPTX 70 kb) Additional file 2:
**Details of antihypertensive drugs and** **anti-dyslipidemia** **drugs used for subjects in study 2.** (PPTX 71 kb)

## Abbreviations

OHA: Oral hypoglycemic agent
CVD: Cardiovascular disease
T2DM: Type-2 diabetes mellitus
DPP-4: Dipeptidyl peptidase-4
ICD: International Classification of Diseases
MI: Myocardial infarction
BG: Biguanide
SU: Sulfonylurea
α-GI: α-glucosidase inhibitor
TZD: Thiazolidinedione
GLP-1: Glucagon-like peptide-1
MHLWJ: Ministry of Health, Labour, and Welfare in Japan
HR: Hazard ratio
