# Supplementary material for: Reduced vascular events in type 2 diabetes by biguanide relative to sulfonylurea: study in a Japanese Hospital Database
Source: BMC Endocr Disord. 2015 Sep 17;15:49. doi: 10.1186/s12902-015-0045-y (PMC4574461; doi:10.1186/s12902-015-0045-y)
Supplement: Additional file 1: — Details of antihypertensive drugs and anti-dyslipidemia drugs used for subjects in study 1. (PPTX 70 kb) [file 12902_2015_45_MOESM1_ESM.pptx]

## Slide 1
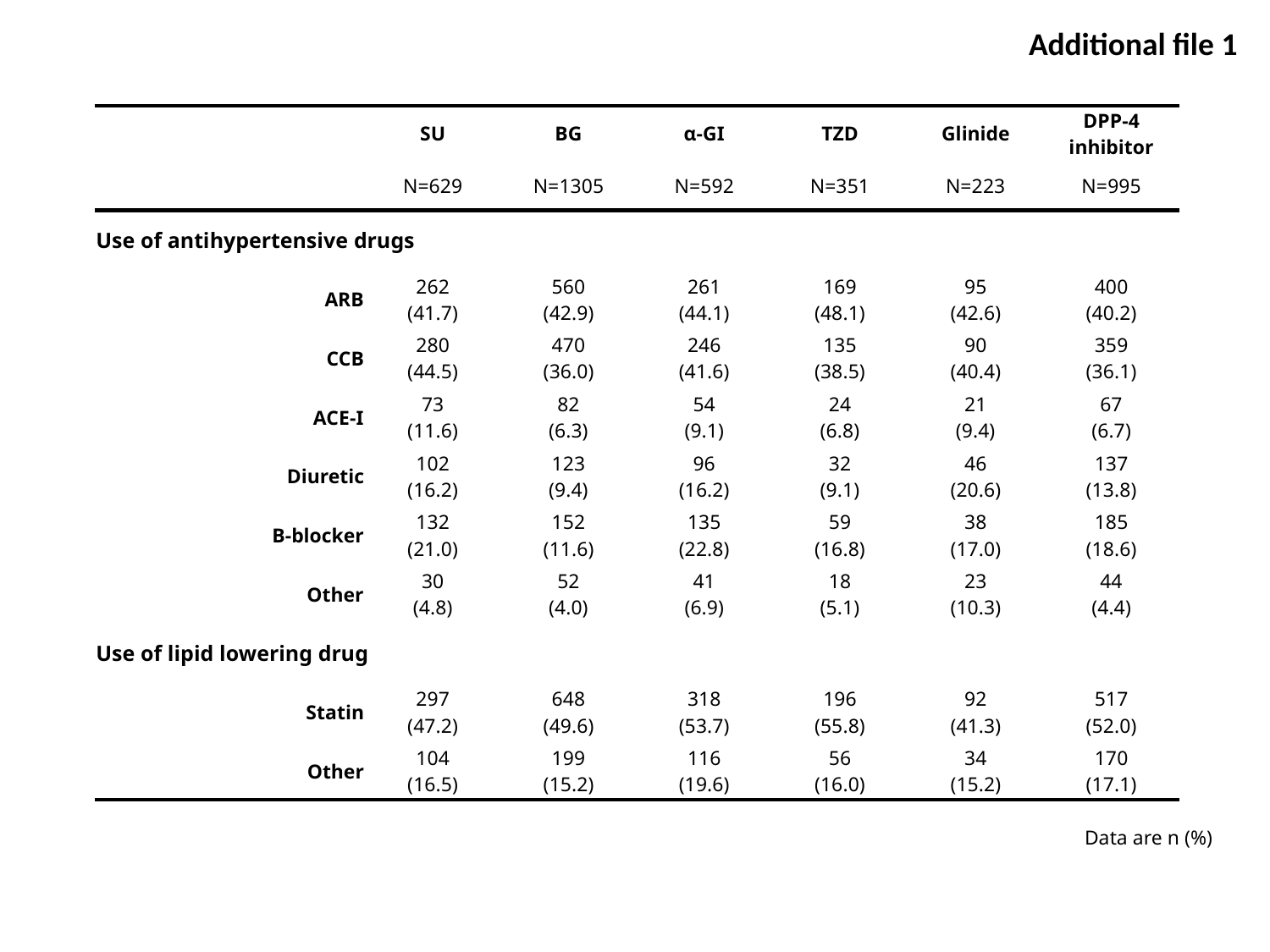

Additional file 1
| | SU | BG | α-GI | TZD | Glinide | DPP-4 inhibitor |
| --- | --- | --- | --- | --- | --- | --- |
| | N=629 | N=1305 | N=592 | N=351 | N=223 | N=995 |
| Use of antihypertensive drugs | | | | | | |
| ARB | 262 (41.7) | 560 (42.9) | 261 (44.1) | 169 (48.1) | 95 (42.6) | 400 (40.2) |
| CCB | 280 (44.5) | 470 (36.0) | 246 (41.6) | 135 (38.5) | 90 (40.4) | 359 (36.1) |
| ACE-I | 73 (11.6) | 82 (6.3) | 54 (9.1) | 24 (6.8) | 21 (9.4) | 67 (6.7) |
| Diuretic | 102 (16.2) | 123 (9.4) | 96 (16.2) | 32 (9.1) | 46 (20.6) | 137 (13.8) |
| Β-blocker | 132 (21.0) | 152 (11.6) | 135 (22.8) | 59 (16.8) | 38 (17.0) | 185 (18.6) |
| Other | 30 (4.8) | 52 (4.0) | 41 (6.9) | 18 (5.1) | 23 (10.3) | 44 (4.4) |
| Use of lipid lowering drug | | | | | | |
| Statin | 297 (47.2) | 648 (49.6) | 318 (53.7) | 196 (55.8) | 92 (41.3) | 517 (52.0) |
| Other | 104 (16.5) | 199 (15.2) | 116 (19.6) | 56 (16.0) | 34 (15.2) | 170 (17.1) |
Data are n (%)
