# Supplementary material for: Reduced vascular events in type 2 diabetes by biguanide relative to sulfonylurea: study in a Japanese Hospital Database
Source: BMC Endocr Disord. 2015 Sep 17;15:49. doi: 10.1186/s12902-015-0045-y (PMC4574461; doi:10.1186/s12902-015-0045-y)
Supplement: Additional file 2: — Details of antihypertensive drugs and anti-dyslipidemia drugs used for subjects in study 2. (PPTX 71 kb) [file 12902_2015_45_MOESM2_ESM.pptx]

## Slide 1
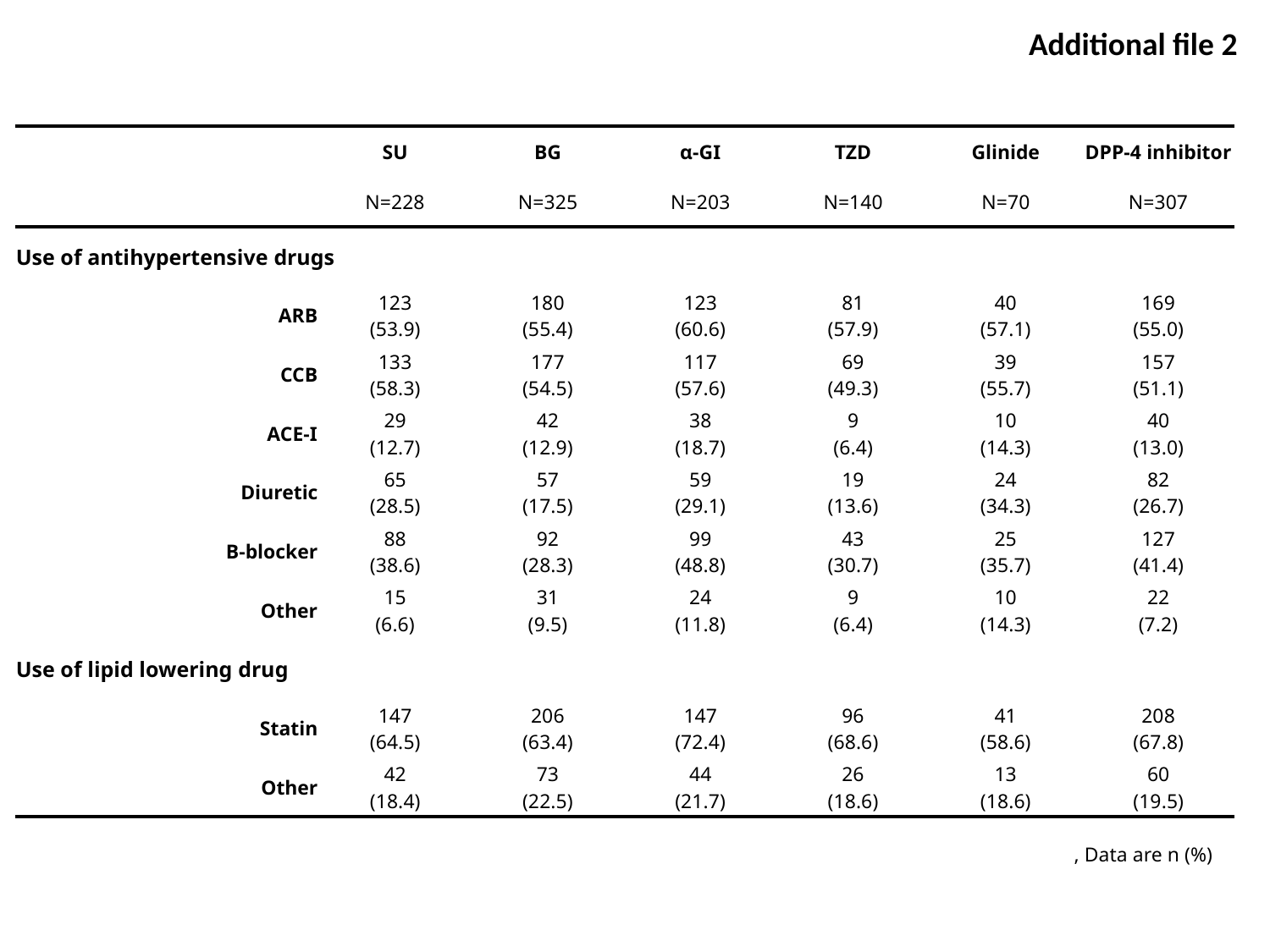

Additional file 2
| | SU | BG | α-GI | TZD | Glinide | DPP-4 inhibitor |
| --- | --- | --- | --- | --- | --- | --- |
| | N=228 | N=325 | N=203 | N=140 | N=70 | N=307 |
| Use of antihypertensive drugs | | | | | | |
| ARB | 123 (53.9) | 180 (55.4) | 123 (60.6) | 81 (57.9) | 40 (57.1) | 169 (55.0) |
| CCB | 133 (58.3) | 177 (54.5) | 117 (57.6) | 69 (49.3) | 39 (55.7) | 157 (51.1) |
| ACE-I | 29 (12.7) | 42 (12.9) | 38 (18.7) | 9 (6.4) | 10 (14.3) | 40 (13.0) |
| Diuretic | 65 (28.5) | 57 (17.5) | 59 (29.1) | 19 (13.6) | 24 (34.3) | 82 (26.7) |
| Β-blocker | 88 (38.6) | 92 (28.3) | 99 (48.8) | 43 (30.7) | 25 (35.7) | 127 (41.4) |
| Other | 15 (6.6) | 31 (9.5) | 24 (11.8) | 9 (6.4) | 10 (14.3) | 22 (7.2) |
| Use of lipid lowering drug | | | | | | |
| Statin | 147 (64.5) | 206 (63.4) | 147 (72.4) | 96 (68.6) | 41 (58.6) | 208 (67.8) |
| Other | 42 (18.4) | 73 (22.5) | 44 (21.7) | 26 (18.6) | 13 (18.6) | 60 (19.5) |
, Data are n (%)
